# Psychological Stress as a Determinant of Increased Maximum Voluntary Bite Force - A Clinical Observational Study

**DOI:** 10.7759/cureus.46106

**Published:** 2023-09-28

**Authors:** Sulthan Ibrahim R Khan, Ghaida Aljammaz, Lama A Alosail, Azzam Almeshrafi, Anupama Ramachandran, Salman Siddeeqh, Abdulmohsen Alfadley

**Affiliations:** 1 Department of Restorative and Prosthetic Dental Sciences, King Saud bin Abdulaziz University for Health Sciences, Riyadh, SAU; 2 Endodontics, King Abdulaziz Medical City - National Guard Health Affairs, Riyadh, SAU; 3 Periodontics, King Abdulaziz Medical City - National Guard Health Affairs, Riaydh, SAU; 4 Periodontics, King Abdullah International Medical Research Centre, Riyadh, SAU; 5 Conservative Dentistry and Endodontics, Chettinad Dental College and Research Institute, Chennai, IND; 6 Maxillofacial Surgery and Diagnostic Sciences, King Saud bin Abdulaziz University for Health Sciences, Riyadh, SAU; 7 Endodontics, Restorative and Prosthetic Dental Sciences, King Saud bin Abdulaziz University for Health Sciences, Riyadh, SAU

**Keywords:** stress, maximum voluntary bite force, psychological stress., perceived stress scale questionnaire, bite force

## Abstract

Background

Psychological stress is a known risk factor and modulator for several oral diseases. It is among the critical etiological factors of bruxism and several other disorders. The quantum of bite force is one of the prime determinants of wear resistance and the clinical performance of restorations. Thus, the present study aims to investigate the relationship between the maximum voluntary bite force (MVBF) and the amount of perceived stress experienced by an individual.

Materials and methods

Patients (n=111) fulfilling the exclusion and inclusion criteria were divided into high, medium, and low-stress groups based on their stress scores deduced from the Perceived Stress Scale questionnaire (PSS). Bite force measurement was recorded in Newtons (N) for each subject using a portable customized bite recording FlexiForce sensor (B 201). The data were analyzed using Kruskal-Wallis and independent samples t-test.

Results

Among the females, the bite force in the medium and high-stress groups was greater compared to the low-stress level group. On the other hand, there was no difference in bite force between any of the stress level groups among male participants.

Conclusion

The results show that higher MVBF is associated with higher perceived stress scores in adult females.

Practical Implications

Psychological counseling can be included in the dental treatment plan of individuals with a high-stress score to counteract their stress-related higher occlusal forces, parafunctional jaw movements, and risky oral health behaviors, thereby potentially reducing the incidence of adverse outcomes such as temporomandibular joint dysfunction and restoration failure by careful choice of restorative materials.

## Introduction

Stress is defined as a process in which the surrounding habitat conditions strain an organism's adaptive capacity, resulting in both psychological demands as well as biological changes that could place the organism at risk for illness [1]. Any hostile condition to a person is defined as a stressor, and the response to a stressor is called a stress reaction. Although stress responses have evolved as an adaptive process, Selye, one of the pioneers in the field of stress psychology, stated that profound chronic stress responses may cause cellular dysfunction and pathology [2]. Psychological stress is ubiquitous in life, causing a response in the brain, which can result in the activation of numerous physiological responses in the body, like the immune, endocrine, and central nervous systems [3,4].

Several studies have shown that chronic stress in humans has been known to influence the course of severe neurological disorders and cardiac problems, gastric ulcers, asthma, diabetes, headaches, and accelerated aging and is also among the causes of premature death [5-7]. Chronic stress can be one of the factors which can initiate or aggravate oral pathology [8]. Research has also demonstrated that psychological stress is a risk factor for bruxism, dental caries, and periodontal diseases [9, 10]. It contributes to the modulation of oral diseases through immune system dysfunction, increased stress hormones, cariogenic bacterial counts, and poor oral health behaviors [10-12].

The bite force is an important criterion influencing the functional state of the masticatory system. It has been shown that the quantum of bite force is one of the determinants of wear resistance and the clinical performance of restorations [13,14]. Wear of the teeth, commonly found in stress-related outcomes like bruxism, is associated with increased bite force [15].

Our literature search revealed that there had yet to be a clinical study on the association between perceived stresses and the quantum of maximum voluntary bite force (MVBF), where the effect of confounders was negated. Therefore, the objective of the present study was to investigate the above premise in clinically asymptomatic individuals.

## Materials and methods

Once the ethical committee approval was obtained (Approval no: RC19/244/R) from the Institutional Review Board (IRB) of King Abdullah International Medical Research Center (KAIMRC), patients visiting the College of Dentistry clinics at King Saud Bin Abdulaziz University for Health Sciences and fulfilling the selection criteria were considered for the study. As there are no previous similar studies, a sample size number of 108 patients was determined based on the sample size calculation assuming a conventional small effect size (difference in the mean MVBF values between the test groups) of 0.24 at 0.05 level of significance to achieve a power of 80% using the G* power software statistical tool.

A purposive sampling technique was used to select the participants of the study. One hundred eleven patients of Arab ethnicity (60 males, 51 females) were included in the study. The sex of participants was defined based on their self-report during the study registration process. Subjects aged 20-45 years with complete natural dentition (excluding third molars), bilateral Angle class I molar, and canine relationship were selected for the study. Subjects with fixed prostheses and large occlusal restorations in first molar teeth (i.e., occlusal restorations extending more than 1/3 of inter-cuspal distance), endodontically treated first molar teeth, active periodontal disease, temporomandibular joint disorders (TMD), previous orthodontic treatment, major psychological and neurological disorders, and systemic diseases were excluded from the study.

After obtaining informed consent, recruited subjects were given the Perceived Stress Scale (PSS) questionnaire (Appendix 1) to fill out, and their stress scores were calculated with the help of a clinical psychologist. The PSS questionnaire contains ten items and is widely used for evaluating stress levels in bruxism and temporomandibular joint dysfunction (TMD) research [16,17]. The subjects were divided into high-, medium-, and low-stress groups based on their stress scores. As there are no normative PSS scores for the studied population, the first quartile (Q1) represents the low-stressed group with a PSS score of 0 to 13, the second (Q2) and third quartiles (Q3) represent the moderately stressed group with a PSS score of 14 to 27. The fourth quartile (Q4) represents a high-stress group with a PSS score of 28 to 40. A similar methodology was previously adopted by Tavalocci et al. [18]

The different stress group subjects were then further divided into subgroups based on sex. Following this, MVBF measurements were recorded in a single session for each subject using a FlexiForce ELF system (Tekscan, Norwood, Massachusetts). This system consists of Flexiforce B201Sensors and ELF Data Acquisition System. Flexiforce is a thin, piezoresistive sensor that produces a measurable change in electronic resistance under occlusal load. This sensor requires prior calibration and can measure bite force values up to 4448 Newtons.

The bite force input in Newtons (N) captured in the sensor is processed and displayed on the computer screen using the ELF data acquisition system, which consists of USB analog to digital ELF handle and software.

Clinical procedure

Two general dentists were involved in bite force recording procedures, and two other dentists were simultaneously involved in data extraction from the computer screen. Bite force recordings were done bilaterally in the first molar region within a few seconds of maximal clenching. The measurements were recorded three times in intervals of about one minute between each measurement to avoid fatigue on each side. The highest value of the maximal voluntary bite force (MVBF) among the six measurements was recorded and subsequently used for statistical analysis.

Statistical analysis

Descriptive and inferential statistics were analyzed by SPSS version 20.0 (IBM Inc., Armonk, New York). Mean and standard deviation were used to summarize the clinical parameters. As the data were non-normally distributed, the Kruskal-Wallis test was used for intergroup comparison of bite force between males and females across the varying stress levels. Dunn-Bonferroni post hoc test was used for multiple pairwise comparisons of bite force across the varying stress levels. Independent samples t-test was used to compare MVBF and stress scores across varying stress categories among male and female groups. A p-value of <0.05 was considered statistically significant. In case statistically significant differences in MVBF were detected among the three different stress groups within the same sex, a contrast correlation analysis was performed.

## Results

Table 1 shows the gender distribution of the recruited study participants among different stress score groups. The average mean age of study participants was 26 years, and there was no difference in age between the gender groups (Table 2).

**Table 1:** Gender distribution of the study participants between different stress score (PSS) groups PSS - Perceived Stress Scale

Stress score (PSS) groups	Gender	Number of study participants (n)	Percentage of distribution of study participants (%)
Low	Female	16	53.3
	Male	14	46.7
Medium	Female	23	53.5
	Male	20	46.5
High	Female	12	31.6
	Male	26	68.4
Overall	Female	51	45.9
	Male	60	54.1

**Table 2:** Gender age analysis using independent samples t-test A p-value of <0.05 was considered statistically significant

Total (N)		Variable	Gender	Mean age	Standard deviation	p-value
111		Age	Male (n=60)	27.03	8.53	0.147
			Female (n=51)	24.78	7.52	
	Overall age			26	8.12180	

Comparison of MVBF between genders at different levels of stress score (PSS) groups showed that males had significantly higher MVBF than females at all stress score levels (Table 3).

**Table 3:** Comparison of Mean biteforce (MVBF) between Gender at different levels of Stress Score (PSS) using independent samples t test. The Bite force is represented as Mean (M) and Standard Deviation (SD) PSS - Perceived Stress Scale, p-value of < 0.05 was considered statistically significant

Stress Score (PSS) Groups	Gender	Mean MVBF (M)	Std.Deviation (SD)	P value
Low	Female	184.56	73.06	0.001
	Male	289.93	62.26	
Medium	Female	228.91	52.72	0.001
	Male	292.55	62.41	
High	Female	253.67	50.63	0.001
	Male	319.15	55.50	

Comparison of stress scores between genders at all levels of stress score (PSS) groups show that the stress scores of females are significantly higher than males in medium and high-stress score groups (Table 4)

**Table 4:** Comparison of mean stress score (PSS) between genders at different levels of stress score groups using independent samples t-test PSS - Perceived Stress Scale A p-value of <0.05 was considered statistically significant

Stress score (PSS) groups	Gender	Mean stress score	Standard deviation	p-value
Low	Female	10.13	2.63	0.980
	Male	10.00	2.25	
Medium	Female	21.04	3.56	0.029
	Male	18.55	3.32	
High	Female	33.25	3.08	0.032
	Male	29.38	2.97	

In females, the MVBF in the low-stress score group was lesser than the other two stress score groups. However, there is no significant difference in MVBF between medium and high-level stress score groups. Comparison of MVBF among males between different stress score (PSS) groups shows that there was no significant difference in bite force between any of the stress score groups (Table 5).

**Table 5:** Comparison of mean bite force (MVBF) among females and males between different stress score (PSS) groups using the Kruskal-Wallis test PSS - Perceived Stress Scale p-value of <0.05 was considered statistically significant

Gender	Stress score (PSS) groups	Number	Mean bite force	Standard deviation	p-value
Female	Low	16	184.56	73.06	0.002
	Medium	23	228.91	52.72	
	High	12	253.67	50.63	
Male	Low	14	289.93	62.26	0.129
	Medium	20	292.55	62.41	
	High	26	319.15	55.50	

As no significant difference was seen among the male groups, a retrospective power analysis was done, and it was found that the study had adequate power. Dunn-Bonferroni post hoc test for multiple pairwise comparisons showed higher MVBF in the high and medium-stress score group compared to the low-stress score group in females (Table 6).

**Table 6:** Dunn-Bonferroni post hoc test for multiple pair comparisons between different stress score (PSS) groups in females PSS- Perceived Stress Scale A p-value of <0.05 was considered statistically significant

Pair comparisons: stress score (PSS) groups	Mean bite force difference	Standard error	p-value
				Low - medium	-12.64	4.84	0.009
Low - high	-19.6	5.67	0.001
Medium - high	-6.96	5.29	0.188

Contrast correlation analysis was performed on the female groups, which showed higher MVBF in the high-stress score group compared to medium and low-stress score groups combined. Also, the bite force was higher in the medium-stress score group than in the low-stress score group (Table 7).

**Table 7:** Contrast study done for a significant ANOVA between different stress score groups among females PSS- Perceived Stress Scale A p-value of <0.05 was considered statistically significant

Variable		Contrast between different stress score (PSS) groups	Bite force mean difference	Standard error	t-test	p-value (2 tailed)
Bite force	Assume equal variances	High PSS vs. medium and low PSS combined	46.93	19.69	2.38	0.021
		Medium PSS ss. low PSS	44.35	19.34	2.29	0.026

## Discussion

The primary aim of this study is to explore the association between MVBF levels and the perceived stress experience of an individual in a clinically asymptomatic adult population. It is widely known that stress initiates the release of various central neurotransmitters, which include serotonin, adrenaline, and dopamine [19]. Animal studies have found positive links between oral behavioral patterns and stress-induced variations of dopamine neurotransmission [20].

The impact of central influences in producing rhythmic orofacial activity in mastication has been established [21]. Hence, chewing is mediated by local factors and effectively controlled by central factors [22]. Psychological stress is associated with an increased chewing frequency, and chewing is an autonomic behavior in response to stressful conditions, potentially contributing to stress-coping mechanisms [23].

The bite force is one of the indices of the functional condition of masticatory apparatus resulting from cranio-maxillary biomechanics [24]. Many supplementary aspects affect the bite force in an individual, including craniofacial anatomy, age, sex, periodontium, temporomandibular joint dysfunction (TMD), and bruxism [25]. Although there are many studies on the above parameters, to the best of our knowledge, no literature has evaluated the influence of a central factor like psychological stress on the maximum voluntary bite force (MVBF). In this study, the inclusion and exclusion criteria were structured to control the effect of various confounding factors like bruxism and temporomandibular dysfunction, which could impact the intensity of MVBF.

As the sex of the patient could be a critical determinant of the variations in stress experience and MVBF, its effect was examined. Results of the current study showed that males have higher MVBF when compared to females at all levels of PSS score groups. This is in accordance with previous studies and is attributed to the fact that males have prominent muscular anatomy and strength compared to females [26-28]. The anatomical characteristic of the masseter muscle in males is type II muscle fibers with more thickness and cross-sectional area than that of females, which could be a contributing factor to the increased MVBF [29]. Another possible explanation is the overall size and surface area of teeth and periodontal ligament area in females are smaller compared to males, which could lead to a lower MVBF [30].

In this study, women had higher stress scores than men among medium and high-stress score groups. Our results are similar to previous studies, which reported women having a greater lifetime prevalence of stress [31-34]. This could be due to personality differences between males and females, in which females express their anxieties and are more vocal about their mental health [35]. Men and women also differ in the manner they participate in cognitive, self-conscious, and referential processes, which in turn may contribute to their differential subjective stress experiences [36]. Thus, it can be seen the sex of the patient has a confounding effect on the stress experience and MVBF. Hence, in the current study, the association of perceived stress and MVBF was analyzed separately among male and female participants.

Our study found that MVBF in females was increased in higher stress score groups compared to the lower stress score group. This finding is consistent with a previous study in which higher perceived stress levels were associated with increased masticatory muscle function when compared to lower stress levels [37, 38]. High stress is more commonly associated with frequent chewing [39] and binge eating [40], which can lead to hyperactivity of masticatory muscle [41] and, consequently, higher bite force values [42].

Furthermore, the increase in MVBF may also be attributed to the strength of the underlying jawbone, which Wolff's law can explain [43]. It states that "bone in a healthy person or animal will adapt according to the loads under which it is placed". Therefore, if loading on a particular bone increases, the bone becomes denser and more robust, and the converse is also true. Thus, due to frequent chewing, a highly stressed person can develop a denser and tougher underlying jawbone and may generate higher MVBF.

Chronically stressed patients are more commonly associated with generalized anxiety disorder, which can potentially result in hyperactivity of the temporalis muscle [44-46]. These overactive temporalis, masseter, and other masticatory muscles can generate higher bite force.

Despite the findings of the association between PSS and MVBF among female PSS score groups, no such correlation was observed among male PSS score groups. Velly et al. [47] found that women were almost three times more likely to develop myofascial pain than men. Generally, stress-related disorders have a higher incidence in women than in men [48, 49]. Stress-related research has shown differences between sexes on both the molecular and whole systems levels, which can be responsible for the increase in endocrine, emotional, and arousal responses to stress in females when compared to males [50].

The possible explanation for why our study subjects were clinically asymptomatic despite high-stress experience can be their relatively young age. However, chronic stress can make them vulnerable to pathologies because of imbalances in homeostasis maintenance and changes in the endocrine, autonomic, and immune systems during aging [51].

Limitations of the study

Bite force measurement is interconnected with physiological and pathological parameters like age, sex, craniofacial morphology, periodontal support, temporomandibular joint health, and dental status. Although efforts have been taken to diminish the sequelae of these factors by rigorous sample selection, nevertheless, the effect of these confounders cannot be eliminated. Furthermore, the participants of this study were limited to a particular geographic region. Hence, further research should be done to test its reliability among different populations and with an increased sample size.

## Conclusions

The data of this study supports the association between psychological stress and oral health factors, as the oral cavity is found to have high emotional importance. The results show that higher bite force is associated with higher perceived stress scores in adult females. From a clinical point of view, highly stressed individuals are often associated with a higher probability of developing dental pathologies. Therefore, clinical psychologists can be included in the dental team to provide early counseling to mitigate stress-induced parafunctional jaw movements and risky oral health behavior in high-stress individuals. Also, preoperative assessment of bite force and stress level can help formulate a suitable dental treatment plan according to individual patients' unique characteristics that include dental material selection while restoring carious or missing teeth to possibly counteract altered occlusal forces.

## Appendices

**Table 8:** Appendix 1 - Perceived Stress Scale questionnaire PSS - Perceived Stress Scale QUESTIONNAIRE PSS Score Interpretation: ►Scores ranging from 0-13 are grouped as low perceived stress. ►Scores ranging from 14-27 are grouped as moderate perceived stress. ►Scores ranging from 28-40 are grouped as high perceived stress.

For each question, choose from the following alternatives: 0 - never 1 - almost never 2 - sometimes 3 - fairly often 4 - very often
Question 1: In the last month, how often have you been upset because of something that happened unexpectedly?
Question 2: In the last month, how often have you felt that you were unable to control the important things in your life?
Question 3: How often have you felt nervous and stressed in the last month?
Question 4: In the last month, how often have you felt confident about your ability to handle your problems?
Question 5: How often have you felt that things were going your way in the last month?
Question 6: In the last month, how often have you found that you could not cope with everything you had to do?
Question 7: In the last month, how often have you been able to control irritations in your life?
Question 8: How often have you felt that you were on top of things in the last month?
Question 9: In the last month, how often have you been angered because of things that happened that were outside of your control?
Question 10: In the last month, how often have you felt difficulties were piling up so high that you could not overcome them?
